# Pathogen Box screening for hit identification against *Mycobacterium abscessus*

**DOI:** 10.1371/journal.pone.0195595

**Published:** 2018-04-26

**Authors:** Jinsun Jeong, Guehye Kim, Cheol Moon, Hyun Jung Kim, Tae Ho Kim, Jichan Jang

**Affiliations:** 1 Division of Applied Life Science (BK21plus Program), Gyeongsang National University, Jinju, South Korea; 2 Department of Clinical Laboratory Science, Semyung University, Jecheon, South Korea; 3 Division of Life Science, Research Institute of Life Science, Gyeongsang National University, Jinju, South Korea; Institute for Integrative Biology of the Cell, FRANCE

## Abstract

*Mycobacterium abscessus* is a rapidly growing life-threatening mycobacterium with multiple drug-resistance mechanisms. However, there is no official regimen for *M*. *abscessus* therapy. In this study, we screened the Pathogen Box, which contains 400 drug-like molecules active against neglected diseases, to identify active molecules targeting *Mycobacterium abscessus* using resazurin live/dead assays. In this screening assay, the Z-factor was 0.7, as an indicator of the statistical confidence of the assay. A cut-off of 80% growth inhibition in the screening resulted in the identification of four different compounds at a single concentration (20 μM). Dose-response curves identified three different hit candidates, i.e., MMV688508, MMV688844, and MMV688845, which generated good inhibitory curves. All hit candidates were expected to have different molecular targets. Among them, MMV688844 showed the best minimum inhibitory concentration value for not only wild-type *M*. *abscessus* but also for nine different R and S morphotype clinical isolates. Thus, we found that MMV688844, identified from the Pathogen Box screen, may be a promising candidate in the *M*. *abscessus* drug discovery pipeline.

## Introduction

*Mycobacterium abscessus* is a potentially life-threatening, pathogenic, rapidly growing mycobacterium (RGM) with multiple intrinsic and extrinsic drug-resistance mechanisms [1]. For example, *M*. *abscessus* contains a thick and waxy mycobacterial cell envelope, similar to other mycobacteria, preventing the penetration of toxic drugs and consequently resulting in poor drug effectiveness. In addition, the *M*. *abscessus* genome contains many genes involved in drug-efflux systems, such as members of the major facilitator family, ABC transporters, and MmpL proteins [2]. *M*. *abscessus* also possesses enzymes possibly involved in natural resistance via the modification and inactivation of antibiotics, such as rifampicin ADP-ribosyltransferase and a mono-oxygenase that may be involved in resistance to rifampicin [2]. An inducible erythromycin ribosome methyltransferase *erm* (41) (MAB_2997), which attenuates clarithromycin and erythromycin activity, was also recently discovered [3]. Extrinsically, *M*. *abscessus* is equipped with acquired antibiotic resistance through spontaneous mutations of molecular targets of antibiotics. Mutations in *rplC* (encoding 50S ribosomal protein L3) are involved in the acquisition of resistance to oxazolidinones [4]. Moreover, *rrl* and the 16S rRNA gene (*rrs*) provide acquired resistance to aminoglycosides and macrolides in *M*. *abscessus* [5, 6].

*M*. *abscessus* can cause skin and severe lung diseases, which progressively advance to illness or fatality. This microorganism may be responsible for the dissemination of infections in immunocompromised patients and in patients who have undergone lung transplantation and treatment for cystic fibrosis (CF) [7]. To determine the relative distributions of different nontuberculous mycobacteria (NTMs) per continent and country, a global survey was conducted with 62 centers from 30 countries across six continents. In this survey, RGM accounted for 50% and 28.7% of isolates, particularly in Taiwan and South Korea, respectively. In Taiwan and South Korea, *M*. *abscessus* and *M*. *fortuitum* were the most frequently isolated RGM after *M*. *avium* complex (MAC) [8]. Furthermore, based on a recent study, approximately 5%–10% of patients with CF are infected with *M*. *abscessus*, and that number continues to grow. Patients with CF show defects in normal mucus production due to mutations in the CF transmembrane conductance regulator (*CFTR*) gene, which encodes a transporter protein. Mucus-filled lungs can easily become infected by pathogens such as *M*. *abscessus*, resulting in the worsening of disease symptoms or even death [9]. Interestingly, a recent study using matrix-assisted laser desorption/ionization time-of-flight mass spectrometry genotyping reported that *CFTR* gene variations, particularly Q1352H, may increase susceptibility to NTM-related lung disease in the Korean population [10]. In addition, another study reported that *M*. *abscessus* accounts for more than 50% of all isolated NTMs, with rates of up to 13% and 16% in patients with CF in Europe and the USA [8].

There are two different morphotypes of *M*. *abscessus*, the rough (R) and smooth (S) forms, which can be distinguished by their surface-associated glycopeptidolipid (GPL) content. The major difference between these two morphotypes is total loss of GPL in the R form [11]. Although both morphotypes have been identified in human airways, the R morphotype tends to be much more virulent and is involved in chronic colonization of the airways in CF patients. In addition, the R morphotype is more pro-inflammatory than the S morphotype. Intravenous (i.v.) *M*. *abscessus* infection models show much more lethal and higher levels of induced tumor necrosis factor-α with the R morphotype than the S morphotype for C57BL/6 mice [12].

However, there is no official regimen for *M*. *abscessus* therapy, particularly for pulmonary infections. Antibiotic treatment outcomes depend on subspecies and geographical location, and monotherapies often fail to produce a microbiological cure [13]. For example, *M*. *abscessus* has different macrolide susceptibilities based on subspecies. *M*. *abscessus* is divided into three separate species or subspecies: *M*. *abscessus* subsp. *abscessus*, *M*. *abscessus* subsp. *massiliense*, and *M*. *abscessus* subsp. *bolletii* [14]. *M*. *abscessus* subsp. *massiliense* has an inactive *erm* gene that confers inducible macrolide resistance, whereas *M*. *abscessus* subsp. *bolletii* and *M*. *abscessus* subsp. *abscessus* have active *erm* genes; all three subspecies are presumed to be the same *M*. *abscessus* subspecies based on 16S rRNA sequences [1]. Thus, combination chemotherapy is required for *M*.* abscessus* infections. The usual cocktail consists of clarithromycin or azithromycin plus amikacin and one of the following: fluoroquinolone, imipenem, doxycycline, or linezolid for up to 6 months [13]. However, no antibiotic class has been shown to be effective for long-term sputum smear conversion in pulmonary infections. Therefore, novel drugs that can replace the current regimen are urgently needed.

In this study, we screened the Pathogen Box, which contains 400 diverse, drug-like molecules active against neglected diseases, such as ascariasis, Buruli ulcer, Chagas disease, and malaria, in order to identify hit compounds for *M*. *abscessus*. From the screen, we found that MMV688844 was the best candidate with potential for lead optimization, exhibiting the lowest minimum inhibitory concentration (MIC) and strong activity against clinical isolates.

## Materials and methods

### Bacterial culture and compounds

*M*. *abscessus* ATCC 19977 was used in all assays for the assessment of compound susceptibility *in vitro*. Clinical isolates were provided by the Korea Mycobacterium Resource Center (KMRC). *M*. *abscessus* was cultured *in vitro* in 7H9 supplemented with albumin dextrose catalase (ADC), glycerol (0.2%, vol/vol), and Tween 80 (0.05%, vol/vol) and kept in at 37°C and 5% CO_2_. The Pathogen Box was provided by Medicines for Malaria Venture (MMV; https://www.mmv.org) in 96-well plates, containing 10 μL of a 10 mM dimethyl sulfoxide (DMSO) solution of each compound at a final concentration.

### Assay validation and hit screen

The assay was validated using the Z-factor. For this, a series of negative and positive controls were measured in 7H9 medium supplemented with 10% (v/v) albumin–dextrose–saline (ADS) enrichment, 0.2% (v/v) glycerol, and 0.05% (v/v) Tween 80, which was inoculated with *M*. *abscessus* (5 × 10^5^ bacteria per well). A total of 1 μL of 10 μM clarithromycin in DMSO solution was used as a positive control, and 1% of the DMSO solution was used as a negative control. The plate was incubated for 40 h at 37°C. After adding resazurin at 0.02% (wt/vol) in 40 μL per well and incubation for 12 h at 37°C, the plates were analyzed using a SpectraMax® M3 Multi-Mode Microplate Reader (Molecular Devices, CA) at an excitation wavelength of 560 nm and emission wavelength of 590 nm, at which the color of the dye changed from blue to pink and the fluorescence in negative control cultures reached 800 relative fluorescence units (RFU). Resazurin is a common marker used to distinguish between live and dead *Mycobacterium* sp. based on the degree of metabolism. Metabolically active bacteria can reduce blue non-fluorescent oxidized resazurin within an environment of viable cells to resorufin, which is pink and fluorescent [15, 16]. The assay was performed as described above in two separate experiments. To validate the degree of separation, the Z-factor and the percent inhibition of the positive and negative controls were determined using the formula:

$$\text{Z}-factor=1-\frac{3\left(\sigma_{\rho }+\sigma_{n}\right)}{\left|\mu_{\rho }-\mu_{n}\right|}$$

where *σ*_*p*_ and *σ*_*n*_ are the standard deviations of the positive and negative controls, respectively, and *μ*_*p*_ and *μ*_*n*_ are the corresponding mean values. A Z-factor between 0.5 and 1.0 indicates an excellent assay and statistically reliable separation between the positive and negative controls. The Pathogen Box was screened against *M*. *abscessus* at 20 μM. *M*. *abscessus* was seeded in 96-well plates (5 × 10^5^ bacteria per well) and was exposed to each compound for 40 h at 37°C. Medium with bacteria alone was used in the negative control wells. Resazurin was added to each well (0.02%, wt/vol), and the plates were analyzed at an excitation of 560 nm and emission of 590 nm. The percentage of growth inhibition was calculated using the RFU value, as described previously [17].

### Drug susceptibility testing (DST)

The MICs of the hit compounds were determined using twofold serial dilution assays. For MICs against *M*. *abscessus* clinical isolates, nine clinical strains were provided from the Korea Mycobacterium Resource Center (KMRC). Briefly, serial twofold dilutions of the hits were prepared in 7H9 supplemented with ADC, glycerol (0.2%, vol/vol), and Tween 80 (0.05%, vol/vol) in 96-well clear microplates (SPL Inc.) to obtain concentration ranges from 200 to 0.39 μM. *M*. *abscessus* WT and clinical isolates were then added to each well at a final concentration of 5 × 10^5^ bacteria per well. Microplates were incubated at 37°C for 40 h. A change in color from blue to pink was used as an indicator of bacterial growth. Concentrations required to inhibit bacterial growth by 50% (IC50s) were determined by fitting the curves with a sigmoidal dose-response using GraphPad Prism software (version 6.05). Amikacin was used as a reference compound.

### Time-kill assays

An early exponential phase mycobacterial culture (10^7^ cfu/mL) was prepared in 30 mL of 7H9 supplemented with ADC, glycerol (0.2%, vol/vol), and Tween 80 (0.05%, vol/vol). For time-kill kinetics assay, cefoxitin and MMV688844 were added at final concentrations of 2.0 and 0.2 mg/mL, respectively, and then serial twofold dilutions of each sample were prepared using the mycobacterial culture. All cultures were grown in Corning® 125-mL Polycarbonate Erlenmeyer Flasks with Vent Caps (Product #431143) at 30°C under shaking conditions (100 rpm) as described previously [18]. To perform cfu counting, 100 μL samples were taken from each bottle, and tenfold dilutions of samples were then made in PBS (900 μL PBS, 100 μL sample) at different time intervals (0, 12, 24, 48, 72, and 96 h). A total of 50 μL of each dilution was plated onto 7H10 agar, containing 10% OADC supplement (Sigma-Aldrich, catalog number: M0678).

## Results and discussion

**Fig 1:**
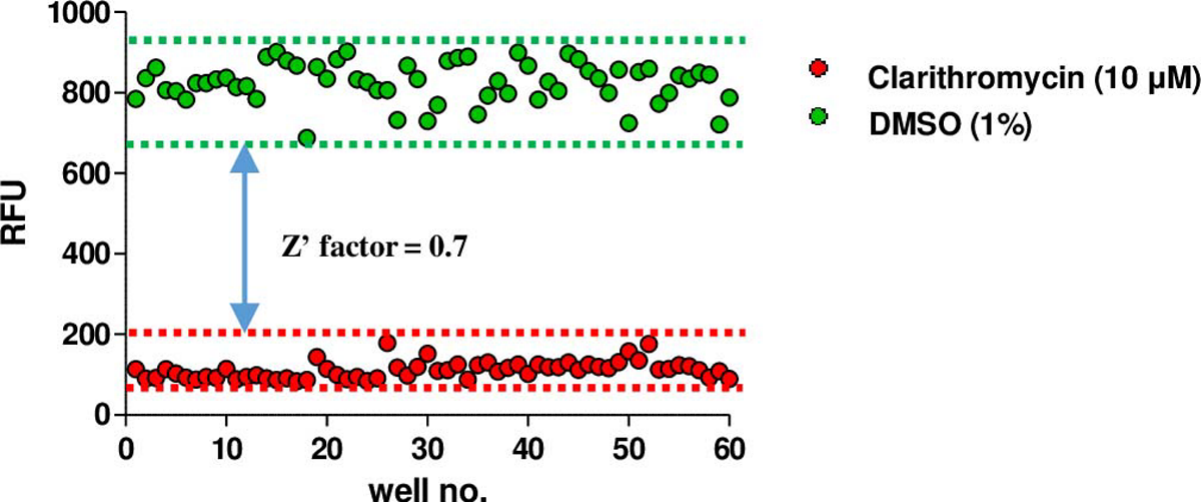
Screening validation. The Z-factor of 0.7 demonstrates excellent assay.

In our study, we identified effective compounds using a screening assay with *M*. *abscessus* grown in the mid-log phase. As shown in S1 Fig., we investigated the growth curve of *M*. *abscessus* to determine the optimal time point for screening. After 28 h of culture, the mid-log phase was observed; this time point was then used for all experiments. For screening, we used resazurin reduction assays. In a previous report, resazurin reduction demonstrated complete correlation with the MICs obtained by cfu assay, especially for *M*. *tuberculosis*, *M*. *bovis* BCG, and *M*. *smegmatis* in a safe, reliable, easy and cost-effective manner [16]. Therefore, we employed the resazurin reduction assay to assess the cell viability of the rapidly growing mycobacterium, *M*. *abscessus*. The quality of the screen was evaluated using the Z-factor based on the percent inhibition against *M*. *abscessus* between the 1.0% DMSO and 10 μM clarithromycin-treated groups as the negative and positive controls, respectively. Fig 1 shows the scatter-plot distribution of the percent inhibition for 1.0% DMSO and 10 μM clarithromycin. The average Z-factor between the 1.0% DMSO negative control and 10 μM clarithromycin-treated positive control in the 96-well test plates was 0.7, indicating that the assay could reliably separate positive and negative controls. These findings supported the feasibility of our drug screening assay for use in *M*. *abscessus* screening.

**Fig 2:**
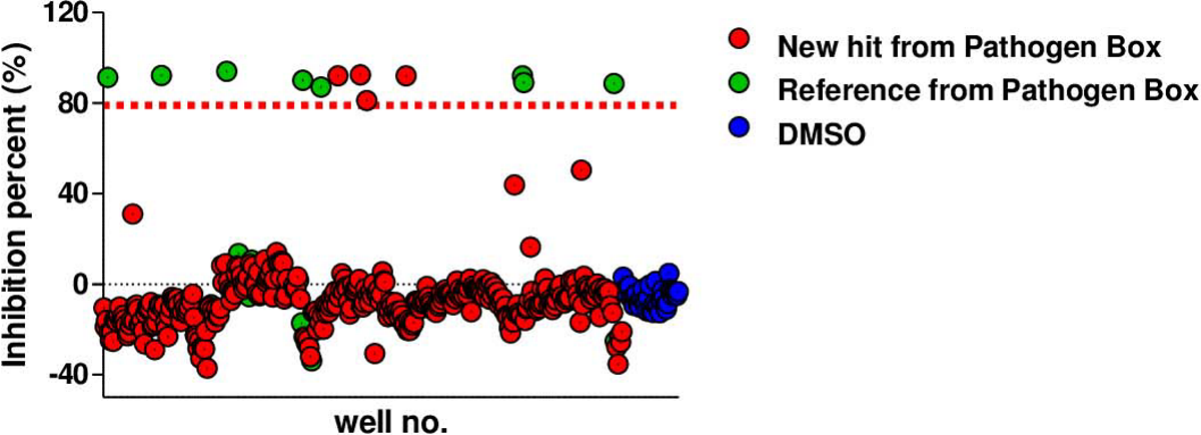
Screening of the Pathogen Box library for *Mycobacterium abscessus*. Scatter plot distribution showing the results of the *M*. *abscessus* screening of the Pathogen Box library using resazurin reduction assay. A total of 400 compounds from the Pathogen Box were screened at 20 μM against *M*. *abscessus*. Growth inhibition of at least 80% was defined as cut-off which resulted in 4 hits (1.0% hit rate) from the 400 compounds in the Pathogen Box library.

The screen was conducted using a one-point concentration (20 μM) of the test compounds. Growth inhibition of at least 80% was defined as the cut-off, yielding four hits (1.0% hit rate; MMV687146, MMV688508, MMV688844, and MMV688845) from the 400 compounds in the Pathogen Box library (Fig 2). As expected, reference compounds that were already included in the Pathogen Box, such as bedaquiline, clofazimine, linezolid, levofloxacin, ofloxacin, rifampicin, radezolid, sutezolid, and auranofin, showed strong inhibition at 20 μM. Other reference compounds that were effective against neglected diseases, such as pentamidine, mefloquine, benznidazole, and miltefosine, showed no activity against *M*. *abscessus* in this screen. Therefore, we concluded that our screen exhibited confidence for the identification of hits from the library.

**Fig 3:**
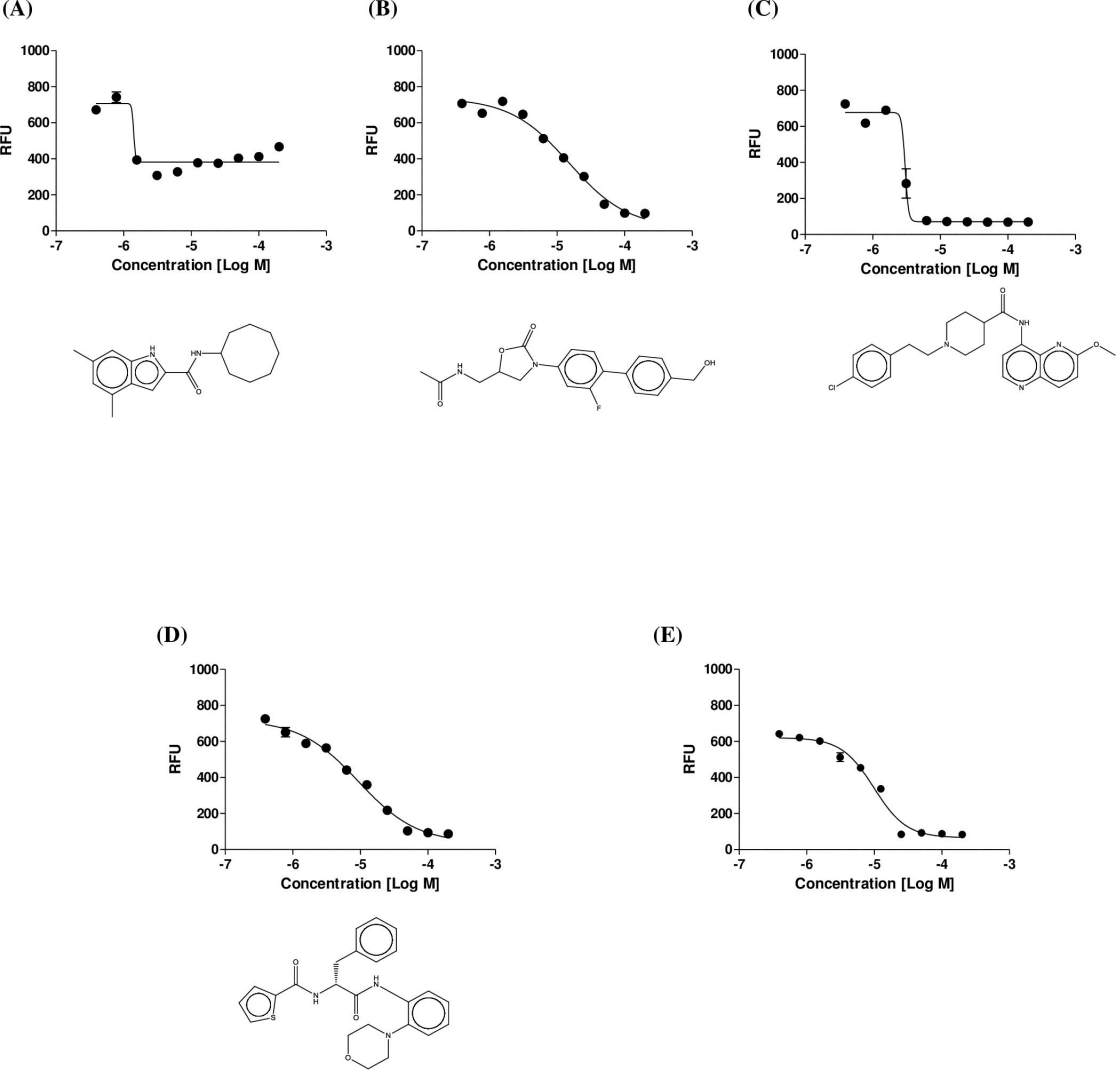
Dose response curve (DRC) and chemical structure of hits. Dose response curve of MMV687146 (A), MMV688508 (B), MMV688844 (C), and MMV688845 (D) and reference compound (Amikacin; E) were plotted and chemical structure of each compounds were described.

The ability of the hits from the screen to inhibit the growth of *M*. *abscessus* was confirmed using a dose-response experiment with resupplied compounds provided by MMV. The MIC_50_ values were 1.4, 15, 2.6, and 9.3 μM for MMV687146, MMV688508, MMV688844, and MMV688845, respectively. Fig 3 shows the dose-dependent inhibitory effects of hit compounds with regard to their activities against *M*. *abscessus*. Of the four compounds with confirmed hits, only three, i.e., MMV688508, MMV688844, and MMV688845 (see chemical structures in Fig 3), displayed dose-dependent activity against *M*. *abscessus*. Although MMV687146 showed the most potent MIC_50_, this hit failed to reduce the RFU value at a high concentration, in contrast to the other hits. MMV687146 exhibited an RFU of more than 400 at the highest concentration in triplicate experiments. However, active compounds, such as MMV688508, MMV688844, and MMV688845, showed complete bacterial growth inhibition at the highest concentration. We assumed that MMV687146 failed to kill or inhibit bacterial survival completely at a high concentration, although this compound showed the best MIC_50_ value. Thus, we decided to omit MMV687146 from our hit list. Amikacin was used as a positive control (MIC_50_ = 9.8 μM).

Of our selected hits, MMV688508 is an intermediate in the synthesis of radezolid, a novel oxazolidinone antibiotic used for the treatment of multidrug-resistant (MDR) infections; this compound has been assessed in two phase II clinical trials [19]. Additionally, linezolid, the first oxazolidinone antibacterial agent, has been applied to the treatment of NTMs, including *M*. *abscessus* [20, 21], and has shown effectiveness in the treatment of chronic and extensively drug-resistant tuberculosis [22]. However, the efficacy of linezolid against NTM still varies among different derivatives, and the clinical use of linezolid in patients with NTM can result in adverse events, such as myelosuppression, in the patient [23]. Thus, MMV688508 may have applications in the treatment of antibiotic-resistant *M*. *abscessus* without side effects, meeting the currently unmet need for safer oxazolidinone agents for the treatment of *M*. *abscessus*. Radezolid was also effective against *M*. *abscessus* in our screen as a reference compound (MMV688327). With regard to molecular targeting, known target genes *rrl* (encoding 23S rRNA) and *rplC* (encoding 50S ribosomal protein L3) have been reported to have acquired resistance to oxazolidinones [24]. MMV688508 showed an MIC of 15 μM in our dose-response experiment.

Second, MMV688844 (TCMDC-143649) has been previously released from GSK’s Tres Cantos Antimycobacterial Set (TCAMS-TB) and showed activity against *M*. *bovis* BCG in replicating and non-replicating *M*. *tuberculosis*. To evaluate the possible mechanisms of action and molecular targets of MMV688844, predictive computational biology algorithms with structural similarity and GSK historical biological assay data were used, and the target was predicted to be an ABC transporter (Rv0194) [25]. In our study, MMV688844 showed the most potent inhibitory activity, with an MIC_50_ as low as 2.6 μM.

Lastly, MMV688845 was found to have efficacy against tuberculosis, as numbered in the fueling open-source library (GSK1729177A). These compounds are potent non-cytotoxic H37Rv hits [26]. In addition, GSK1729177A was previously shown to have MIC_90_ values of 50 μM in THP-1 cells and 6.6 μM in H37Rv [27]. In our study, the MIC_50_ of MMV688845 was 9.3 μM against *M*. *abscessus*.

**Fig 4:**
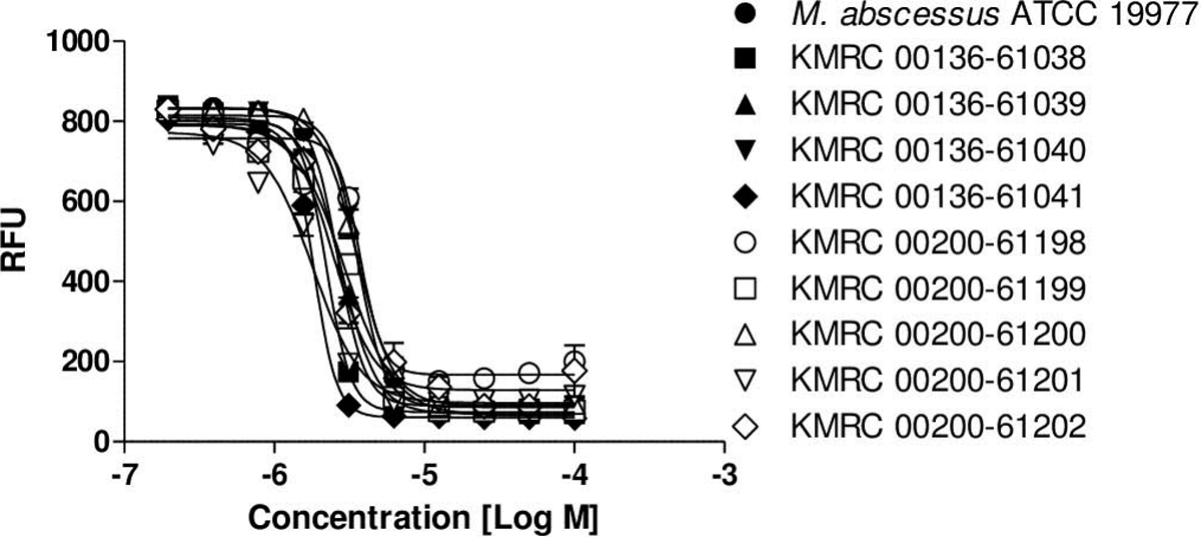
In vitro activity of MMV688844. Activity of MMV688844 against *Mycobacterium abscessus* clinical isolates. Dose-response curves were plotted from REMA data for *M*. *abscessus* strains treated with MMV688844. Data are expressed as the mean ± standard deviation (SD) of triplicates for each concentration. RFU, relative fluorescence units.

*M*. *abscessus* isolates were collected from the Korea Mycobacterium Resource Center (KMRC). We further evaluated the potency of MMV688844 against nine clinical isolates of *M*. *abscessus*, including R and S colony morphotypes. As shown in Fig 4, MMV688844 was equally effective against eight *M*. *abscessus* clinical isolates. MMV688844 even strongly inhibited R-type *M*. *abscessus*, which tends to be much more virulent and is involved in the chronic colonization of CF airways. Thus, optimization of MMV688844 may have the potential to deliver a suitable candidate for preclinical trials.

**Fig 5:**
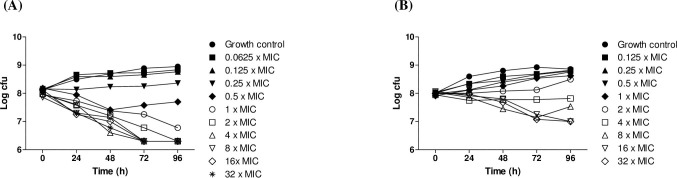
Time–kill curves of (A) cefoxitin and (B) MMV688844 against *Mycobacterium abscessus*. The bacteria were grown in liquid culture (Middlebrook 7H9 medium) in the presence of the indicated concentrations of cefoxitin or MMV688844.

In order to determine whether MMV688844 is bactericidal or bacteriostatic, *M*. *abscessus* was treated with amikacin and MMV688844 using time-kill kinetics. Fig 5 shows the mycobacterial inhibitory pattern of *M*. *abscessus* at different concentrations of the tested antibiotics on different days. In the experiments with cefoxitin, we observed that less than 0.25× MIC did not exhibit an effective killing effect at up to 96 h (Fig 5A) However, cefoxitin killing was observed at concentrations above 1× MIC. Representatively, 2× MIC showed a 2.5 log reduction in comparison with the growth control that was not treated with cefoxitin at 96 h. In addition, no bacterial regrowth was observed even at 1× MIC. In the case of MMV688844, no significant inhibitory effect was observed below 4× MIC (Fig 5B). However, there was a smaller decline in bacterial cfu/mL above 8× MIC in comparison with cefoxitin. For example, MMV688844 showed a 1.8 log reduction compared to the growth control at 16× MIC. Inhibition appeared to be maximized at 16× MIC, and regrowth was observed at 96 h for 8× MIC. However, bacterial regrowth was not observed at 6× or 32× MIC up to 96 h. Based on these results, we conclude that MMV688844 is a bacteriostatic agent.

In summary, we screened the Pathogen Box in a statistically confident screening model and identified several hits in the dose-response curve. Among these hits, MMV688844 showed the best *in vitro* activity against wild-type *M*. *abscessus* and various clinical isolates. MMV688844, which was found to have acceptable cytotoxicity, will be used in further studies as a lead compound in the development of new treatment options for pulmonary *M*. *abscessus* infection. Additional studies will concentrate on the structure-activity relationship, target deconvolution, and *in vivo* efficacy.

## Supporting information

S1 Fig Growth curves of *Mycobacterium abscessus*. (TIF)
