# Supplementary figures and images for: Pathogen Box screening for hit identification against Mycobacterium abscessus
Source: PLoS One. 2018 Apr 26;13(4):e0195595. doi: 10.1371/journal.pone.0195595 (PMC5919404; doi:10.1371/journal.pone.0195595)

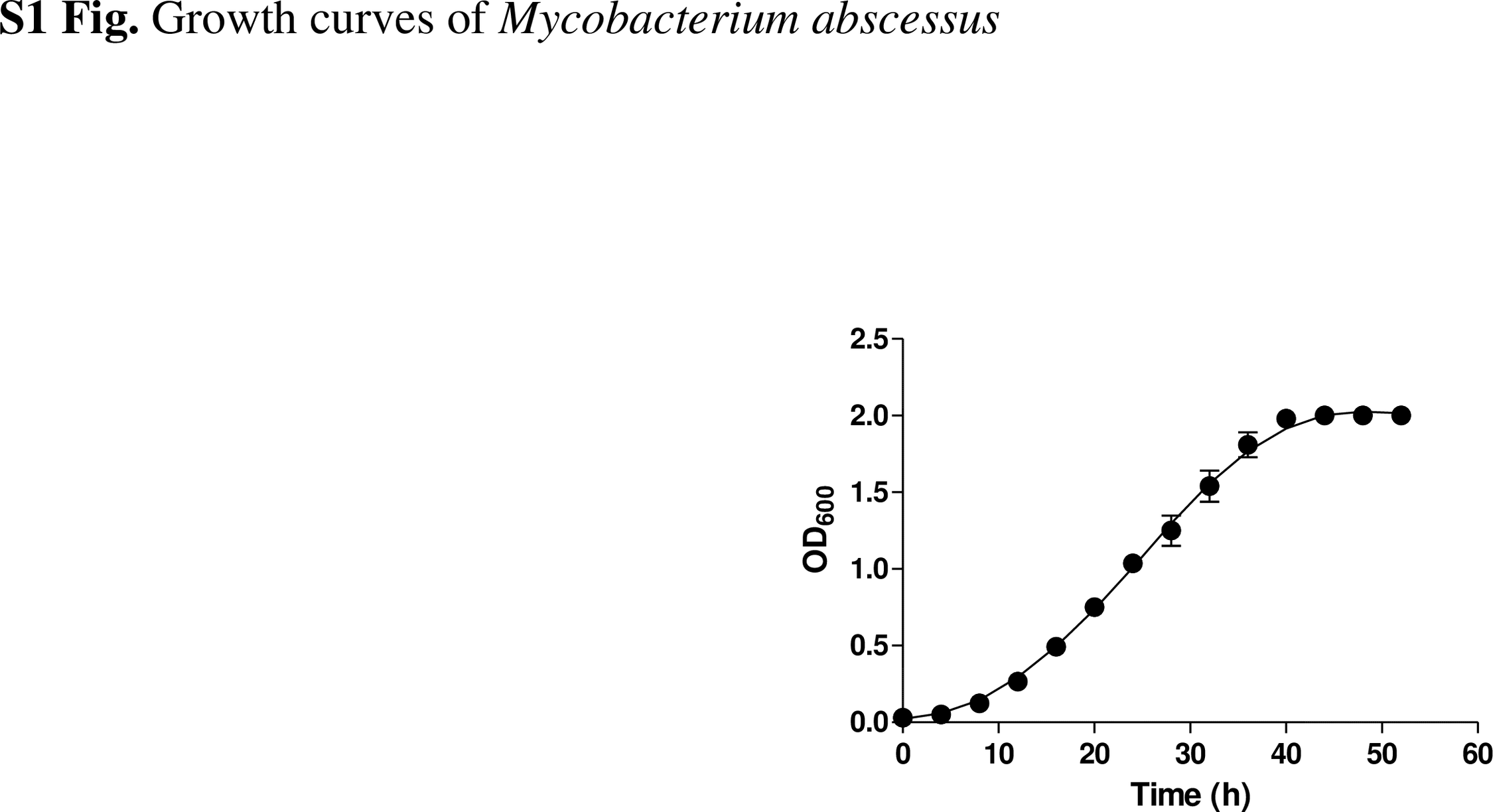

Supplement: S1 Fig — (TIF) [file pone.0195595.s001.tif]
